# Stepwise Translocation of Dpo4 Polymerase during Error-Free Bypass of an oxoG Lesion

**DOI:** 10.1371/journal.pbio.0040011

**Published:** 2006-01-03

**Authors:** Olga Rechkoblit, Lucy Malinina, Yuan Cheng, Vitaly Kuryavyi, Suse Broyde, Nicholas E Geacintov, Dinshaw J Patel

**Affiliations:** **1**Structural Biology Program, Memorial Sloan-Kettering Cancer Center, New York, New York, United States of America; **2**Biology Department, New York University, New York, New York, United States of America; **3**Chemistry Department, New York University, New York, New York, United States of America; Stanford UniversityUnited States of America

## Abstract

7,8-dihydro-8-oxoguanine (oxoG), the predominant lesion formed following oxidative damage of DNA by reactive oxygen species, is processed differently by replicative and bypass polymerases. Our kinetic primer extension studies demonstrate that the bypass polymerase Dpo4 preferentially inserts C opposite oxoG, and also preferentially extends from the oxoG•C base pair, thus achieving error-free bypass of this lesion. We have determined the crystal structures of preinsertion binary, insertion ternary, and postinsertion binary complexes of oxoG-modified template-primer DNA and Dpo4. These structures provide insights into the translocation mechanics of the bypass polymerase during a complete cycle of nucleotide incorporation. Specifically, during noncovalent dCTP insertion opposite oxoG (or G), the little-finger domain–DNA phosphate contacts translocate by one nucleotide step, while the thumb domain–DNA phosphate contacts remain fixed. By contrast, during the nucleotidyl transfer reaction that covalently incorporates C opposite oxoG, the thumb-domain–phosphate contacts are translocated by one nucleotide step, while the little-finger contacts with phosphate groups remain fixed. These stepwise conformational transitions accompanying nucleoside triphosphate binding and covalent nucleobase incorporation during a full replication cycle of Dpo4-catalyzed bypass of the oxoG lesion are distinct from the translocation events in replicative polymerases.

## Abstract 2

Crystal structures of the bypass polymerase Dpo4 at different stages of lesion-bypass reveal how the cell copes with oxidative DNA damage.

## Introduction

Y-family polymerases are able to bypass a variety of DNA lesions that impede high-fidelity replicative DNA polymerases. Such bypass polymerases exhibit a higher error rate and lower processivity on undamaged DNA templates, and can extend from mismatched base pairs (reviewed in [1,2]). Studies suggest that translesion Y-family DNA polymerases are temporarily recruited to overcome blocks to replicative polymerases [3,4]. Y-family polymerases have more spacious and solvent-accessible active sites as observed for archaeal DNA polymerase IV (Dpo4) [5] and Dbh [6], yeast pol η [7], human pol ι [8], and pol κ [9], crystallized in the apo form (pol η, Dbh, pol κ), and as ternary complexes with an incoming deoxyribonucleotide triphosphate (dNTP) (Dpo4, pol ι). The solvent-accessible nature of the active site and the smaller number of contacts of the template-primer DNA with the polymerase enable Dpo4 to accommodate unusual DNA structures in its active site. These include frameshift-template misaligned sequences [5], the *cis–syn* thymine photodimer [10], a bulky benzo[*a*]pyrene-diol–epoxide-adenine lesion [11], an abasic site [12], a reverse wobble G•T mismatch [13], and an ethenoguanine lesion [14]. Structural studies have elucidated the effect of metal ions, nucleotide selection, and pyrophosphorolysis on Dpo4 fidelity [15].

By contrast, replicative polymerases produce tight-fitting, solvent-excluding active sites upon binding of the correct nucleotide; the O helix in the finger domain undergoes a large movement to position itself on the flat surface of a complementary, Watson-Crick nascent base pair [3,16]. This may represent a kinetic rate–limiting step that occurs prior to covalent nucleotide incorporation which has been interpreted in terms of an induced-fit mechanism [17]. If an unusual DNA alignment or a damaged base is encountered at the active site, the O helix often remains in the “open,” inactive conformation [18–21]. In striking contrast to the replicative polymerases, the finger domains of Y-family Dpo4, Dbh, pol η, pol ι, and pol κ polymerases are missing the equivalent of the O helix [8,9,22]. Instead, the replicating base pair is contacted by a β-sheet (Dpo4 residues 41–46) of the finger domain, which forms the rigid roof of the active site, and by an adjacent extended loop (Dpo4 residues 53–59). Kinetic studies indicate that the Y-family pol η and Dpo4 employ a rate-limiting protein conformational change before covalent nucleotide incorporation occurs at the active site [23,24]. However, examinations of currently available crystal structures of Y-family polymerases do not reveal any obvious conformational changes, and it has been suggested that bypass polymerases, such as Dpo4, are always in the “closed,” ready-for-catalysis conformation [22,25]. The absence of an open-to-closed conformational transition was also observed in the case of the repair gap–filling pol λ [26]; the dNTP is accommodated in the space that was, in the binary complex, occupied by the side chain of a tyrosine residue, and the template strand is repositioned toward the active site.

7,8-dihydro-8-oxoguanine (oxoG) is the major known product of oxidation of DNA by reactive oxygen species induced by either ionizing radiation, photochemical mechanisms, or normal cellular metabolic activity [27]. An increased risk for developing cancer has been linked to oxidative stress due to the overproduction of reactive oxygen species resulting from the response of cells to inflammation and infection [28]. Replicative polymerases in vitro readily insert C or A opposite the oxoG lesion in varying proportions that depend on the polymerase, with extension occurring preferentially from oxoG•A mispairs [29–31]. In contrast, the Y-family polymerases yeast and human pol η [32], and Dpo4 (this study), preferentially insert C opposite oxoG, and also preferentially extend from the oxoG•C base pair, thus achieving error-free bypass of this lesion.

To date, a number of crystal structures have been reported for replicative polymerases with oxoG-modified template-primers and dNTPs positioned opposite the lesion or the adjacent 5′-template base, corresponding to insertion and extension ternary complexes, respectively. These include insertion ternary complexes, with oxoG positioned opposite 2′-deoxycytidine 5′-triphosphate (dCTP) and 2′-deoxyadenosine 5′-triphosphate (dATP), with the repair gap–filling pol β [33], and opposite dCTP for the replicative polymerases Rb69 [18] and pol T7 [34], as well as extension complexes past oxoG•C and oxoG•A base pairs by pol T7 [34] and *Bacillus* pol I [35].

The objectives of this study are to address a major gap in our understanding of the conformational changes associated with the polymerase translocation steps that occur in Y-family polymerase–DNA substrate complexes that accompany dNTP insertion and the nucleotidyl transfer reaction. We have chosen Dpo4 due to its high homology to human pol η and similar lesion bypass properties, thereby making it a useful model system for studying Y-family polymerases [36]. We report on crystal structures of three complexes of bypass polymerase Dpo4 and oxoG-modified template-primer DNA, thus capturing two crucial steps of the polymerization cycle. Our results highlight novel and unanticipated conformational changes associated with the Dpo4-DNA interface upon dCTP binding opposite oxoG and upon covalent incorporation of this nucleobase into the primer strand, thereby providing snapshots of the intermediate translocation mechanics for a bypass polymerase.

## Results

The objectives of this work were to investigate the conformational changes that occur when a dNTP is inserted opposite an oxoG template base in the active site of Dpo4, and the subsequent incorporation of this nucleobase into the nascent DNA strand. Since translesion bypass of oxidatively damaged DNA templates by Dpo4 has not been previously investigated to our knowledge, we first surveyed the kinetics of translesion bypass of oxoG in order to select the most appropriate dNTP to be inserted opposite this lesion in our subsequent co-crystal structural studies.

### Fidelity of Translesion Synthesis and Primer Extension Kinetics

**Figure 1:**
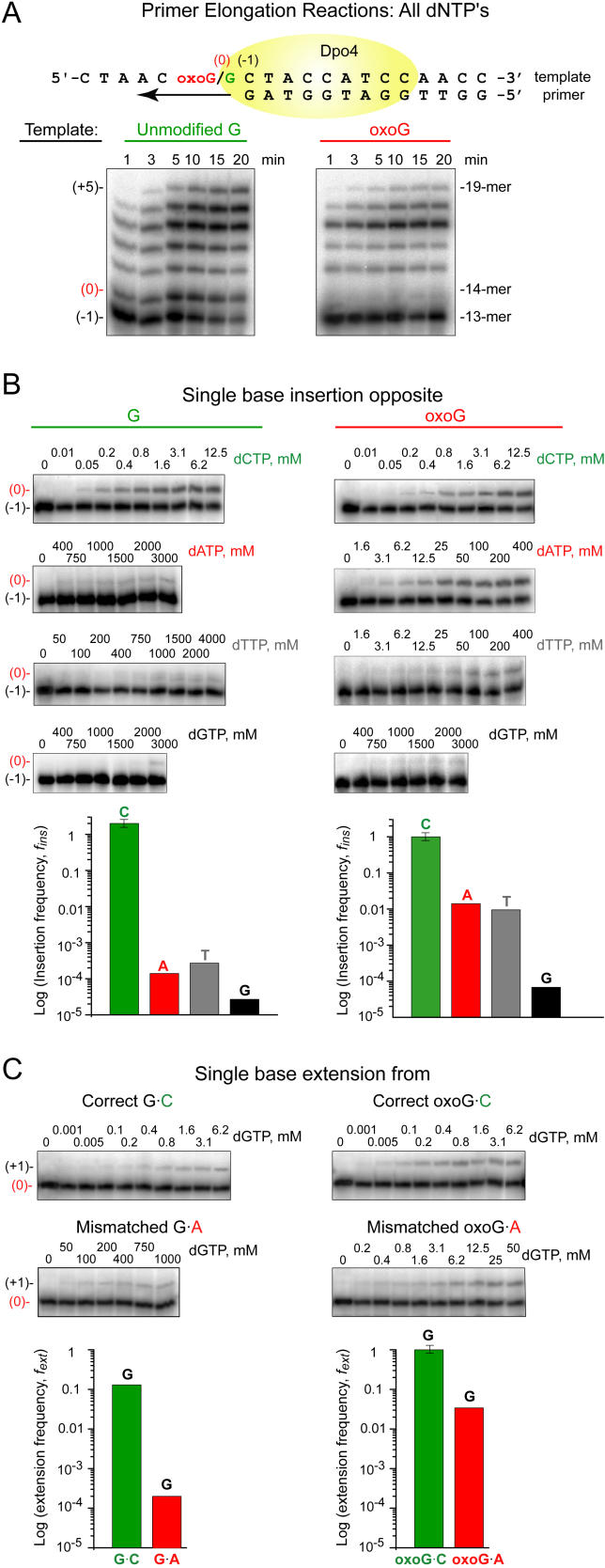
Fidelity and Efficiency of Base Incorporation on Unmodified G and oxoG-Modified Templates by Dpo4 (A) Time course of ^32^P 5′-end-labeled 13-mer primer extension on 19-mer G (left panel) or oxoG (right panel) templates in the presence of all four dNTPs by Dpo4. The labeled (−1) position or 13-mer represents the 13-mer primer with the 3′ end positioned one base before the G or oxoG; the 14-mer extended to position (0) opposite G or oxoG; the 19-mer extended to position (+5) corresponds to full extension. (B) Kinetics of single nucleotide insertion on G (left panel) and oxoG (right panel) templates by dCTP, dATP, dTTP and dGTP. The gels (top panels) show data as a function of dNTP concentration, while the plots (bottom panels) measure the log of the frequency of nucleotide insertion for each dNTP. (C) Kinetics of single nucleotide extension from C and A opposite G (left panel) and oxoG (right panel) templates by the next correct nucleotide (dGTP). The gels (top panels) show data as a function of dGTP concentration, while the plots (bottom panels) measure the log of the frequency of nucleotide extension from G/oxoG•C and G/oxoG•A pairs. The primer extension data recorded under single-hit polymerization conditions (less than 20% of primer extended) were used for estimation of Michaelis-Menten parameters listed in Table 1.

The results of steady-state, one-step primer extension experiments are summarized in Table 1. Primer extension studies in vitro utilizing all four dNTPs show that Dpo4 readily elongates past guanine (G) or oxoG lesions in the template strand to produce full-length, 19-mer extension products (Figure 1A). Michaelis-Menten parameters *V_max_* and *K_m_* (Table 1), were determined for the dNTP insertion step opposite oxoG (or G at the same site) (Figure 1B), and for the one-step extension step beyond an oxoG•C or an oxoG•A base pair (Figure 1C). The insertion frequency, *f_ins_*, of dCTP opposite oxoG is only 2-fold smaller than it is opposite G, with the *f_ins_* values opposite oxoG decreasing in the order dCTP > dATP, dTTP > dGTP (Table 1). Interestingly, the *f_ext_* value is significantly greater for the one-step extension beyond an oxoG•C base pair than beyond a normal G•C base pair at the same site. Also, extension beyond an oxoG•A mispair is ~30 times smaller than extension from an oxoG•C base pair (Table 1). Taken together, these kinetic primer extension data suggest that the oxoG lesion is readily bypassed by Dpo4 in a predominantly error-free manner. In view of our primer extension results catalyzed by Dpo4, we have focused our structural studies on complexes of oxoG-modified template-primer DNA and Dpo4, in the absence and presence of incoming dCTP as well as covalently incorporated C opposite the oxoG lesion site.

**Table 1:**
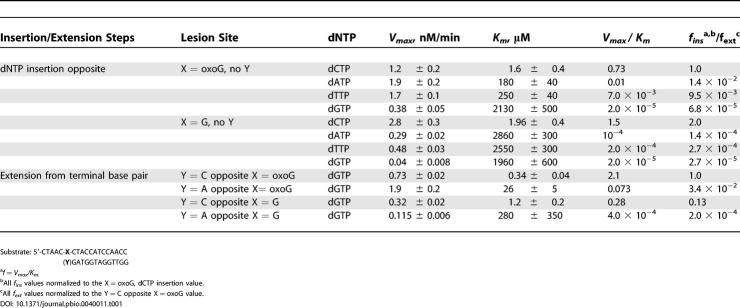
Kinetic Parameters of Insertion and Extension Catalyzed by Dpo4

### DNA Substrate Design and Crystal Structure Determination

**Figure 2:**
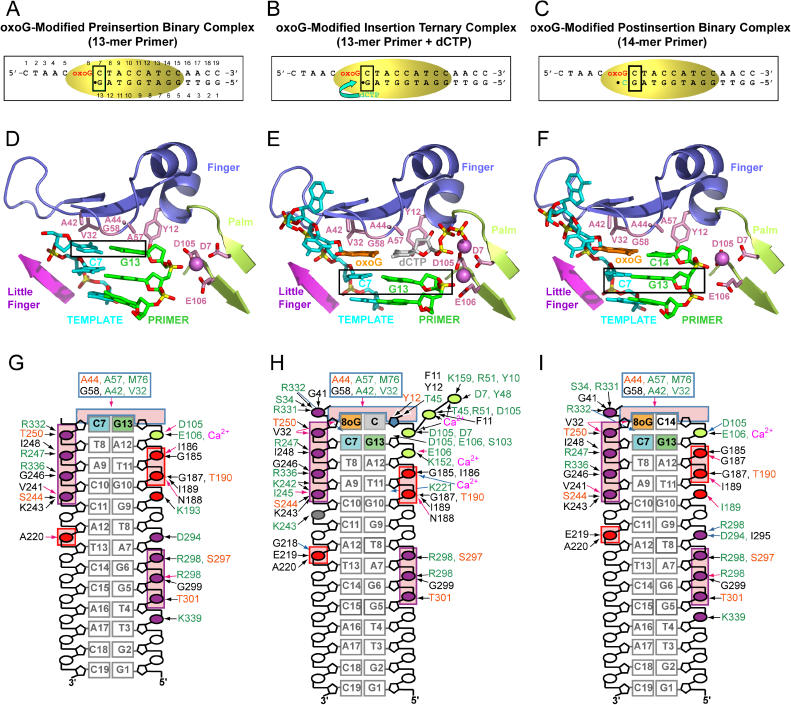
Structures and Intermolecular Contact Details of Dpo4 Complexes with oxoG-Modified Template-Primer DNA (A) Schematic of the pairing of the oxoG-containing 19-mer template strand with the 13-mer primer strand ending in a 2′,3′-dideoxynucleotide in the preinsertion binary complex with Dpo4. (B) Schematic of the pairing of the oxoG-containing 19-mer template strand with the 13-mer primer strand ending in a 2′,3′-dideoxynucleotide plus incoming dCTP in the insertion ternary complex with Dpo4. dCTP is positioned opposite oxoG. (C) Schematic of the pairing of the oxoG-containing 19-mer template strand with the 14-mer primer strand ending in a 2′,3′-dideoxynucleotide in the postinsertion binary complex with Dpo4. Covalently incorporated C is positioned opposite oxoG. (D) Structure of the active site of the preinsertion binary complex. The DNA and protein are in stick and ribbon representations, respectively. Template residues 1–6 (including oxoG) of single-stranded overhang are disordered in the electron density maps. (E) Structure of the active site of the insertion ternary complex, with incoming dCTP paired with oxoG*(anti)* at the active site. (F) Structure of the active site of the postinsertion binary complex, with covalently incorporated C paired with oxoG*(anti).* (G) Details of the intermolecular contacts in the structure of the preinsertion binary complex. (H) Details of the intermolecular contacts in the structure of the insertion ternary complex. (I) Details of the intermolecular contacts in the structure of the postinsertion binary complex. Color-coding for panels G–I. DNA-backbone phosphate groups interacting with the palm domain are shown in light green, the phosphate groups interacting with the thumb domain within the minor groove are shown in red, and the phosphate groups interacting with the little-finger domain within the major groove are shown in purple. The conserved interactions are boxed for the thumb and little-finger contacts. Dpo4 amino acids interacting via the peptide backbone are labeled black, via their side chains, green, and via the backbone and side chains, orange. Black arrows indicate hydrogen bonding, blue arrows water-bridged contacts, and pink arrows depict van-der-Waals contacts.

In order to capture Dpo4 conformational changes during DNA synthesis, we crystallized this bypass polymerase in three types of complexes: the preinsertion binary complex containing oxoG (or G) 19-mer template and 13-mer primer strand (Figure 2A), the insertion ternary complex with a dCTP at the active site paired with oxoG (or G) in the template strand (Figure 2B), and the postinsertion binary complex with the incorporated C residue at the 3′ end of the primer paired with oxoG in the template strand (Figure 2C). All three types of complexes (Figure 2) belong to the P2_1_ space group with distinct unit cell parameters and with two complexes per asymmetric unit (Table 2).

**Table 2:**
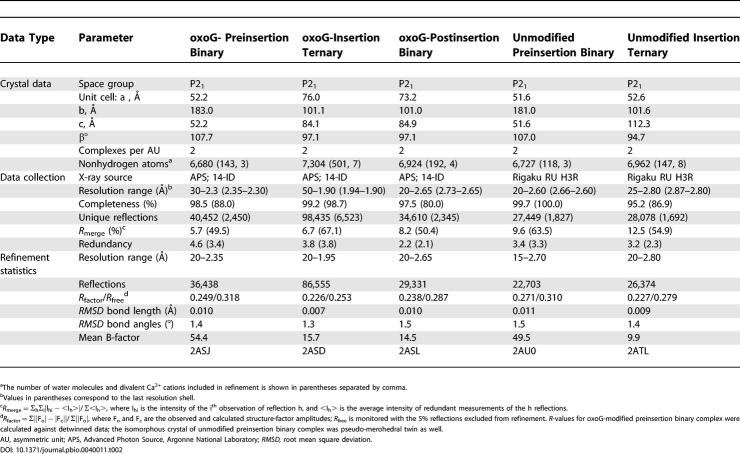
Crystal Data, Data Collection, and Refinement Statistics

The structure of the Dpo4 oxoG-modified insertion ternary complex was solved by molecular replacement employing the published Dpo4 ternary complex with an 18-mer unmodified template–12-mer primer junction and incoming ddADP [5] as a search model, and refined to 1.95-Å resolution. The molecular replacement method was employed to solve the structures of the preinsertion and postinsertion binary complexes using the oxoG-modified insertion ternary complex as a search model. The crystal data, together with the data collection and refinement statistics for all structures, are summarized in Table 2.

### Structure of the oxoG-Modified Preinsertion Binary Complex

We have determined the crystal structure of the Dpo4 preinsertion binary complex with oxoG-modified 19-mer template/dideoxy-terminated, 13-mer primer at 2.35-Å resolution (Figure S1A). The corresponding structure of the preinsertion binary complex with unmodified G was solved at 2.7-Å resolution (Figure S2A). The 5′-C-T-A-A-C-oxoG (or G) single-stranded template overhang is disordered in both crystals. The structures of the oxoG-modified and unmodified preinsertion binary complexes are similar, with root mean square deviation *(RMSD)* = 0.68 Å; therefore we focus on the description of the oxoG-modified complex. The alignment of residues in the Dpo4 polymerase active site pocket is outlined in Figure 2D, with the C7•G13 base pair (the numbering scheme is defined in Figure 2A), adjacent to the oxoG lesion, enclosed in a box for reference. The roof of the active site, formed by the finger domain (in blue), is positioned directly over this C•G pair in the preinsertion binary complex (The Dpo4 bears only one Ca^2+^ cation, coordinated by invariant D7, D105, and E106 residues Figure 2D).

The Dpo4 polymerase embraces the 19-mer template/13-mer primer DNA by its four domains: palm (residues 1–10 and 78–166), finger (residues 11–77), thumb (residues 167–233), and little-finger (residues 244–341) (see Figures S1A and S2A). The thumb is joined to the little-finger domain by a 10-amino-acid-long tether (residues 234–243) that allows positioning of the little finger on the other side of the DNA duplex. The contacts between Dpo4 amino acid residues and the DNA in the oxoG-modified preinsertion binary complex (see Figure S1A) are depicted in Figure 2G. The phosphate groups interacting with the thumb domain within the minor groove are shown in red, and the phosphate groups interacting with the little-finger domain within the major groove are shown in purple, with the conserved interactions boxed for both domains.

### Structure of oxoG-Modified Insertion Ternary Complex with Incoming dCTP

We have determined the 1.95-Å crystal structure of the Dpo4 insertion ternary complex with oxoG-modified, 19-mer template/dideoxy-terminated 13-mer primer, and dCTP positioned opposite oxoG (see Figure S1B). The corresponding structure of the insertion ternary complex with unmodified G was solved at 2.80-Å resolution (Figure S2B). Residues 3–19 of the template strand in the oxoG-modified complex and the entire template strand in the unmodified complex were successfully traced in the electron density map. The structures of the oxoG-containing and unmodified insertion ternary complexes are similar with *RMSD* = 0.60 Å; therefore we focus on the description of the oxoG-modified complex.

**Figure 3:**
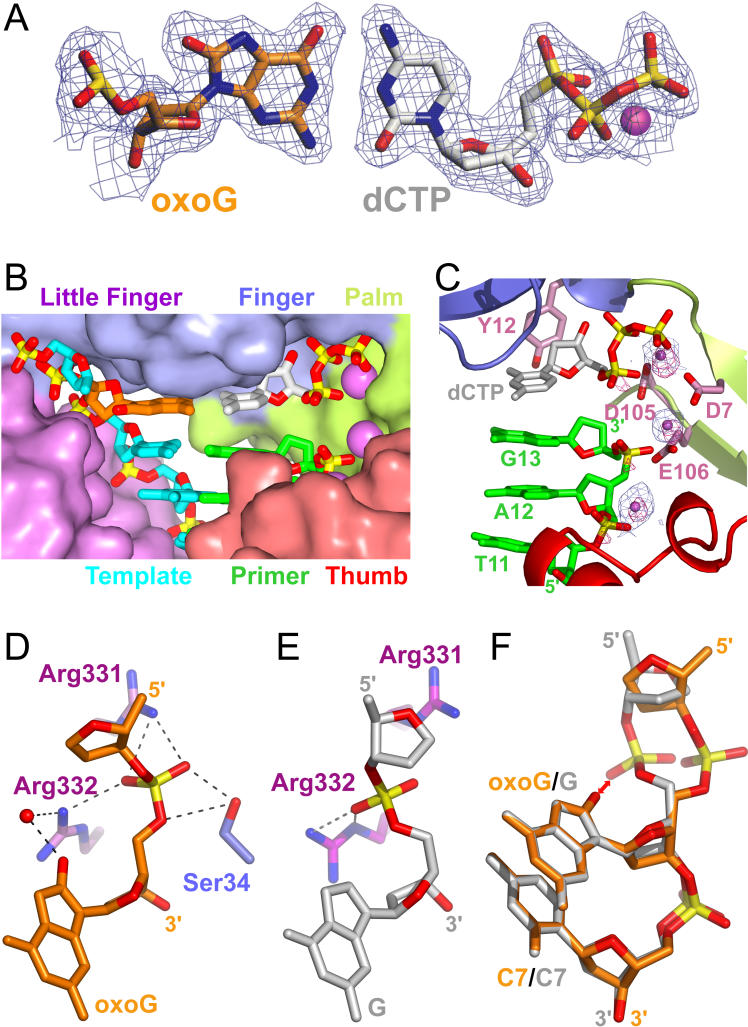
Comparison of Pairing Alignments and Intermolecular Interactions Involving oxoG-Modified and Unmodified G Residues in Insertion Ternary Complexes with Incoming dCTP (A) oxoG*(anti)* paired with dCTP*(anti)* in the oxoG-modified insertion ternary complex, with a coordinated Ca^2+^ cation. 2Fo-Fc 1.95 Å electron density map contoured at 1σ level (1.95 Å resolution). (B) Shape-complementarity between incoming dNTP and the Dpo4 active site pocket containing two chelated Ca^2+^ cations (pink spheres). The dCTP (in silver) is aligned opposite oxoG lesion (in gold). (C) Distinguishing Ca^2+^ from Mg^2+^ ions in the Dpo4 oxoG-modified insertion ternary complex. Strong peaks, contoured in pink at 5 σ level, are found in the 2.0 Å anomalous map plotted for a dataset collected at 1.5418 Å wavelength (Mg^2+^ cation has a much weaker anomalous scattering at this wavelength than Ca^2+^ cation). 2Fo-Fc electron density map for Ca^2+^ cations contoured in blue at 3 σ level are shown by small pink spheres. (D) Interactions between oxoG nucleotide and amino acid side chains of Dpo4 in the oxoG-modified insertion ternary complex. (E) Interactions between G nucleotide and amino acid side chains of Dpo4 in the unmodified G insertion ternary complex. (F) Comparison of the phosphate backbone conformation of C5-oxoG6-C7 (gold) and unmodified C5-G6-C7 (gray) segments within the Dpo4 active site of their respective insertion ternary complexes.

The finger domain, which is on top of the C7•G13 base pair in the active site of the preinsertion binary complex (Figure 2D), is now positioned over the replicating oxoG*(anti)*•dCTP base pair (Figure 3A) in the active site of the insertion ternary complex (see Figure 2E). The side chains of the hydrophobic residues V32 and A42 and the backbone of G58 are packed against the purine ring of oxoG, and the hydrophobic residues A44, A57, and Y12 are packed against the pyrimidine and sugar rings of incoming dCTP. The positioning of the triphosphate backbone of dCTP within the catalytic pocket is shown in Figure 3B. Details of the intermolecular contacts between Dpo4 and the oxoG-containing template-primer junction and the dCTP in the insertion ternary complex (see Figure S1B) are shown schematically in Figure 2H.

Four divalent cation sites were identified in the first molecule, and three sites were found in the second molecule within the asymmetric unit of the oxoG-modified insertion ternary complex. Since the crystallization conditions for the Dpo4-DNA complexes included 100 mM calcium and 5 mM magnesium ions, we used the anomalous signal of the calcium ion at 1.5418 Å (Cu Kα radiation) to distinguish it from the much weaker anomalous scattering by the magnesium ion at this wavelength. Seven strongest peaks on the anomalous map allowed us to assign all ions to calcium. We find one Ca^2+^ ion coordinated by the catalytic triad, the second chelated by the phosphate groups of the incoming dCTP, with the third ion coordinated by the loop of the thumb domain (residues 181 and 186), adjacent to the tip of helix H (Figure 3C). The fourth calcium ion (absent in the second molecule of the asymmetric unit) is bound between adjacent C•G and A•T base pairs in the free portion of the double-stranded DNA region.

### Comparison of oxoG versus G Alignments in Insertion Ternary Complexes

Both oxoG*(anti)*•dCTP (Figure 3A) and G*(anti)*•dCTP pairing alignments are of the Watson-Crick type in their respective insertion ternary complexes. The sugar-phosphate backbone of the oxoG and G residues and their interactions with amino-acid side chains in their respective insertion ternary complexes are shown in Figure 3D and 3E, respectively. Accommodation of the oxoG carbonyl functionality within the Dpo4 polymerase active site results in a shift of the phosphate group of the oxoG base by 3.5 Å and a change in the backbone torsion angle α (O3′-P-O5′-C5′) by 176° as compared with the unmodified structure (superimposed segments compared in Figure 3F), with a similar observation reported previously for the pol β complex [33]. The oxygen atoms of the relocated phosphate group form two hydrogen bonds with the guanidine group of Arg331, one hydrogen bond with the guanidine group of Arg332, and two hydrogen bonds with the hydroxyl group of Ser34 (Figure 3D). The carbonyl oxygen at C8 forms a water-mediated hydrogen bond with the side chain of Arg332, thus helping to lock the oxoG residue in the *anti* conformation (Figure 3D). By contrast, the phosphate group of unmodified G forms only two hydrogen bonds with the guanidine group of Arg332 (Figure 3E); thus oxoG*(anti)* forms four more hydrogen bonds with Dpo4 (Figure 3D) than unmodified G does (Figure 3E).

### The dCTP-Binding Step: Conformational Transitions from Preinsertion Binary to Insertion Ternary Complex

**Figure 4:**
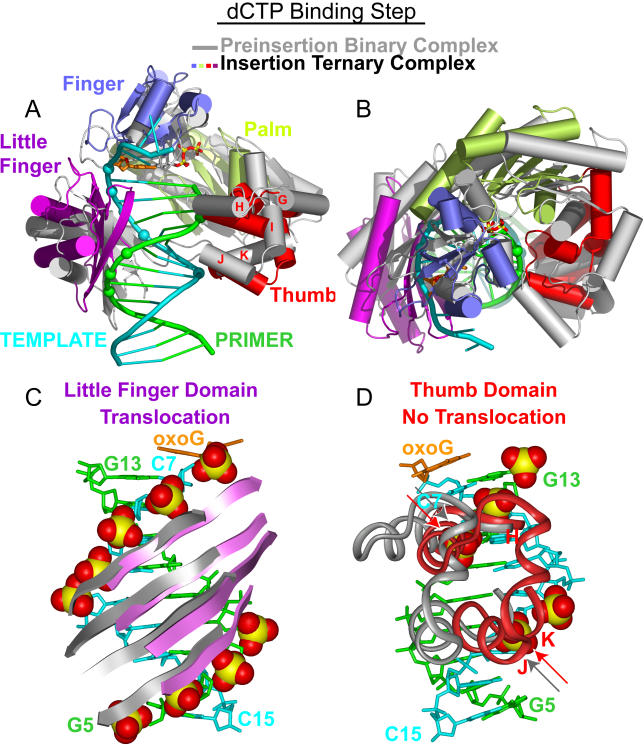
Conformational Transitions of Dpo4 and oxoG-Modified Template-Primer DNA Associated with the Nucleotide-Binding Step Following Superimposition of DNA Duplexes (A) Overall comparative views normal to the DNA helix axis (following superimposition of the DNA duplexes) of the structures of the preinsertion binary complex (in silver) and the insertion ternary complex with incoming dCTP (in color). The DNA-backbone phosphate groups in contact with the Dpo4 little-finger and thumb domains in the ternary complex are shown by colored spheres. (B) Overall comparative view looking down the DNA helix axis. (C) Comparative views of contacts between the little-finger domain and the DNA backbone, with contacts shown by CPK spheres. The β-sheets of the little-finger domain move by a one nucleotide step upon proceeding from preinsertion binary complex (silver) to insertion ternary complex (purple). (D) Comparative views of contacts between the thumb domain and the DNA backbone, with contacts shown by CPK spheres. The α-helical segments of the thumb domain (helixes H, K, J) retain the same contacts with the backbone phosphates of the primer and template strands (shown by arrows) upon proceeding from preinsertion binary complex (silver) to insertion ternary complex (red).

**Figure 5:**
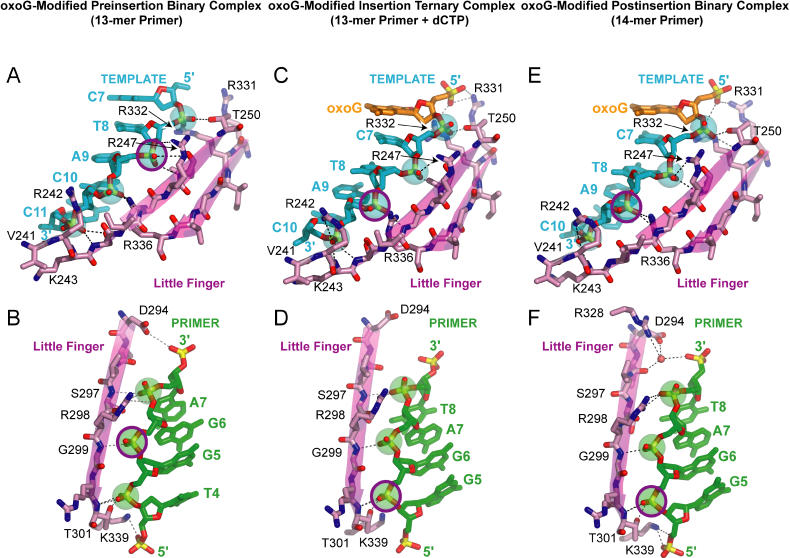
Details of Changes in Dpo4 Little-Finger Domain Contacts with Backbone Phosphates on the oxoG-Modified Template-Primer DNA Associated with the Nucleotide Binding and Incorporation Steps (A) Intermolecular contacts between the little-finger domain (purple) and the template strand (cyan) in the preinsertion binary complex. The phosphate groups interacting with Dpo4 amino acids are highlighted by shaded spheres. The phosphate group of A9 of the template strand is circled for reference. (B) Intermolecular contacts between the little-finger domain (purple) and the primer strand (green) in the preinsertion binary complex. The phosphate groups interacting with Dpo4 amino acids are highlighted by shaded spheres. The phosphate group of G6 of the primer strand is circled for reference. (C) Intermolecular contacts between the little-finger domain (purple) and the template strand (cyan) in the insertion ternary complex. The intermolecular contacts involving the little finger are translocated by one nucleotide step on proceeding from the preinsertion binary complex (in A) to the insertion ternary complex (in C) upon dCTP binding. (D) Intermolecular contacts between the little-finger domain (purple) and the primer strand (green) in the insertion ternary complex. The intermolecular contacts involving the little finger are translocated by one nucleotide step on proceeding from the preinsertion binary complex (in B) to the insertion ternary complex (in D) upon dCTP binding. (E) Intermolecular contacts between the little-finger domain (purple) and the template strand (cyan) in the postinsertion binary complex. The intermolecular contacts involving the little finger are retained on proceeding from the insertion ternary complex (in C) to postinsertion binary complex (in E) on dCTP incorporation. (F) Intermolecular contacts between the little-finger domain (purple) and the primer strand (green) in the postinsertion binary complex. The intermolecular contacts involving the little finger are retained on proceeding from the insertion ternary complex (in D) to postinsertion binary complex (in F) on dCTP incorporation.

**Figure 6:**
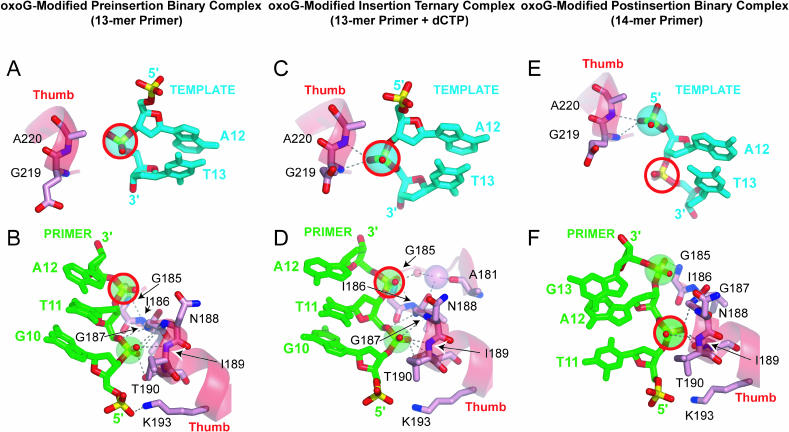
Details of Changes in Dpo4 Thumb Domain Contacts with Backbone Phosphates on the oxoG-Modified Template-Primer DNA Associated with the Nucleotide Binding and Incorporation Steps (A) Intermolecular contacts between the thumb domain (red) and the template strand (cyan) in the preinsertion binary complex. The phosphate groups interacting with Dpo4 amino acids are highlighted by shaded spheres. The phosphate group of T13 of the template strand is circled for reference. (B) Intermolecular contacts between the thumb domain (red) and the primer strand (green) in the preinsertion binary complex. The phosphate groups interacting with Dpo4 amino acids are highlighted by shaded spheres. The phosphate group of A12 of the primer strand is circled for reference. (C) Intermolecular contacts between the thumb domain (red) and the template strand (cyan) in the insertion ternary complex. The intermolecular contacts involving the thumb are retained on proceeding from the preinsertion binary complex (in A) to the insertion ternary complex (in C) on dCTP binding. (D) Intermolecular contacts between the thumb domain (red) and the primer strand (green) in the insertion ternary complex. The intermolecular contacts involving the thumb are retained on proceeding from the preinsertion binary complex (in B) to the insertion ternary complex (in D) on dCTP binding. (E) Intermolecular contacts between the thumb domain (red) and the template strand (cyan) in the postinsertion binary complex. The intermolecular contacts involving the thumb are translocated by one nucleotide step on proceeding from the insertion ternary complex (in C) to the postinsertion binary complex (in E) upon dCTP incorporation. (F) Intermolecular contacts between the thumb domain (red) and the primer strand (green) in the postinsertion binary complex. The intermolecular contacts involving the thumb are translocated by one nucleotide step on proceeding from the insertion ternary complex (in D) to the postinsertion binary complex (in F) upon dCTP incorporation.

We now compare the structures of the preinsertion binary complex with the insertion ternary complex that contains a dCTP molecule opposite oxoG by superpositioning their DNA duplexes in order to identify conformational changes associated with translocation of the polymerase during the dCTP-binding step. This superposition for the entire complex is shown in Figure 4A and 4B, with little-finger domain–DNA interactions shown in Figure 4C and thumb domain–DNA interactions shown in Figure 4D. The key observation is that the finger, palm, and little-finger domains move up by one nucleotide to allow for the noncovalent insertion of dCTP into the active site (Figure 4C, with details shown in Figure 5), while the thumb maintains its contacts with the DNA duplex (shown by arrows in Figure 4D, with details shown in Figure 6).

The little finger slides and rotates counterclockwise (with respect to the 5′-to-3′ direction of the template strand) as a rigid entity along the DNA duplex, resulting in translocation of the polymerase by one step (Figure 4C), thereby allowing the next template base and incoming dCTP to enter the active site. Upon dCTP binding, the contacts of the little finger with the phosphate groups of residues T8, A9, C10, and C11 (Figure 5A) are relocated to the phosphate groups of C7, T8, A9, and C10 (Figure 5C) on the template strand. At the same time, related little-finger contacts on the primer strand are displaced from the phosphate groups of G5, G6, and A7 (Figure 5B) to the phosphate groups of G6, A7, and T8 (Figure 5D). It is interesting that the contacts between the DNA phosphate groups and the little finger, which is unique among Y-family bypass polymerases, involve electrostatic interactions between alternating arginine and peptide backbone nitrogen groups (Figure 5A and 5C). The little finger provides an interface along which the DNA can more easily slide during the translocation step in a manner that is independent of base sequence (see Figure 4C). The little finger is positioned closer to the primer strand in the preinsertion binary complex than in the insertion ternary complex, thus allowing for additional hydrogen-bonding contacts with the phosphate groups of T8 in the template strand (Figure 5A) and G5 in the primer strand (Figure 5B) in the former complex. By contrast, as a result of the dCTP binding, the α-helical segments of the thumb domain (helixes H, K, J) change their relative position with respect to the palm domain, and even though they rotate counterclockwise relative to the DNA, they maintain the same contacts with the phosphate of T13 of the template strand (Figure 6A and 6C) and with the backbone phosphates of T11 and A12 of the primer strand (Figure 6B and 6D).

In quantitative terms, dCTP binding results in rotation of the little finger with respect to the DNA by 29°, almost its full-twist value, while the palm and finger domains, as well as the thumb domain, rotate by 18°, half of their full rotation cycle (Table 3). The little-finger domain, as well as the palm and finger domains, moves along the DNA axis by 3.2–3.5 Å, generating space for nascent base-pair formation. By contrast, the thumb domain, which maintains its contacts with DNA on bonding of dCTP, does not translate with respect to the DNA (Table 3).

**Table 3:**
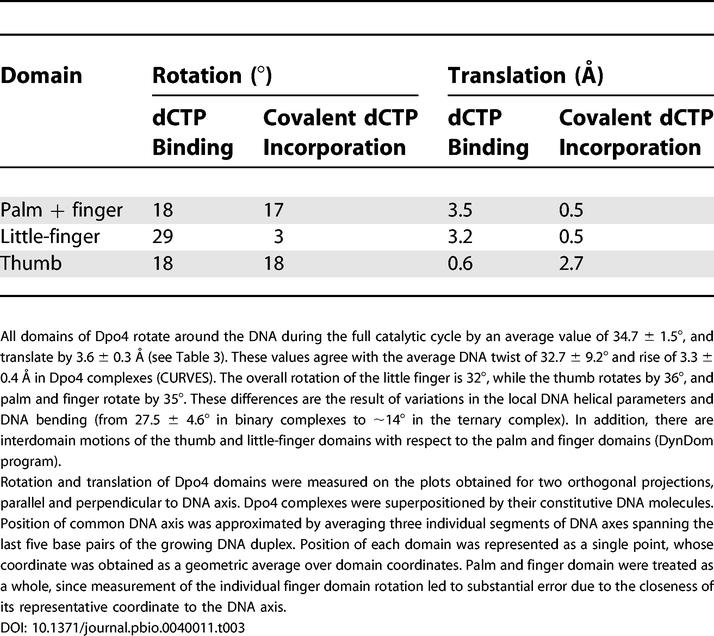
Rotation and Translation of Dpo4 Domains with Respect to DNA for the Preinsertion Binary to Insertion Ternary Step (dCTP Binding) and for the Insertion Ternary to Postinsertion Binary Step (dCTP Incorporation)

### Structure of the oxoG-Modified Postinsertion Binary Complex with Covalently Incorporated Cytosine Opposite oxoG

In the case of the postinsertion binary complex, a covalently incorporated cytosine is positioned opposite the oxoG site (see Figure 2C) and forms a Watson-Crick oxoG•C pair. Residues 3–19 of the template strand can be traced in the electron density map. The finger domain is positioned directly over the Watson-Crick oxoG•C pair with one Ca^2+^ cation coordinated by the D7, D105, and E106 at the active site of Dpo4 (see Figure 2F). Details of the intermolecular contacts between Dpo4 and the oxoG-containing template-primer junction in the postinsertion binary complex (Figure S1C) are shown schematically in Figure 2I.

### The Cytosine-Incorporation Step: Conformational Transitions from Insertion Ternary Complex to Postinsertion Binary Complex

**Figure 7:**
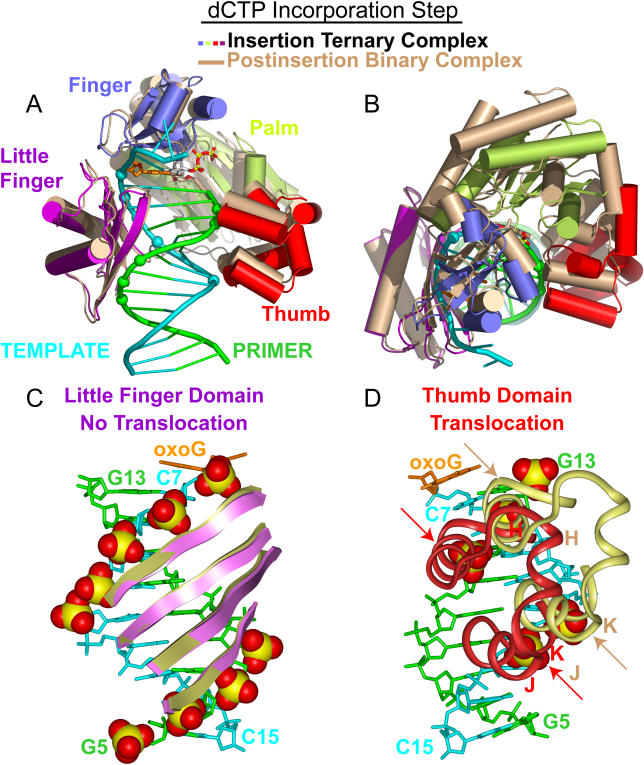
Conformational Transitions of Dpo4 and oxoG-Modified Template-Primer DNA Associated with the Covalent Incorporation Step Following Superimposition of DNA Duplexes (A) Overall comparative views (following superimposition of DNA duplexes) of the structures of the insertion ternary complex with incoming dCTP (in color) with the postinsertion binary complex after covalent cytosine incorporation (in beige). The DNA-backbone phosphate groups in contact with the Dpo4 little-finger and thumb domains in the ternary complex are shown by colored spheres. (B) Overall comparative view looking down the DNA helix axis. (C) Comparative views of contacts between the little-finger domain and the DNA backbone are maintained upon proceeding from the insertion ternary complex (purple) to the postinsertion binary complex (beige). (D) Comparative views of contacts between the thumb domain and the DNA backbone, which shift by a one nucleotide step (shown by arrows) upon proceeding from the insertion ternary complex (purple) to the postinsertion binary complex (beige).

We compare the structures of the insertion ternary complex that contains the incoming dCTP molecule opposite oxoG with the postinsertion binary complex that contains covalently incorporated C opposite oxoG by superpositioning their DNA duplexes in order to identify the conformational changes associated with translocation of the polymerase during the dCTP incorporation step. This superposition for the entire complex is shown in Figure 7A and 7B, with little-finger-domain–DNA interactions shown in Figure 7C and thumb domain–DNA interactions in Figure 7D. The key observation is that the little-finger domain maintains its contacts with the DNA (Figure 7C), while the thumb domain moves by one nucleotide (shown by arrows in Figure 7D) during covalent incorporation of the cytosine opposite the oxoG lesion site. These conformational changes in the thumb domain on cytosine incorporation are accompanied by the release of the pyrophosphate together with a divalent cation.

Upon covalent cytosine incorporation, there is retention of little-finger contacts with the phosphate groups of C7, T8, A9, and C10 on the template strand (see Figure 5C and 5E), and the phosphate groups of G6, A7, and T8 on the primer strand (see Figure 5D and 5F). By contrast, the contact of the thumb domain with the phosphate group of T13 (see Figure 6C) is relocated to the phosphate group of A12 (see Figure 6E) on the template strand. At the same time, related thumb-domain contacts on the primer strand are displaced from the phosphate groups of T11 and A12 (see Figure 6D) to the phosphate groups of A12 and G13 (see Figure 6F).

In quantitative terms, covalent cytosine incorporation results in rotation of the thumb domain and palm and finger domains by 17 to 18 degrees, half of their full rotation cycle, while the little finger undergoes a minimal rotation of 3° (Table 3). The thumb domain moves along the DNA axis by 2.7 Å, while the little-finger domain, which maintains its contacts with DNA on cytosine incorporation, and palm and finger domains, undergo minimal translation (0.5 Å) with respect to the DNA (Table 3).

### dCTP Positioning in Insertion Ternary Complex

**Figure 8:**
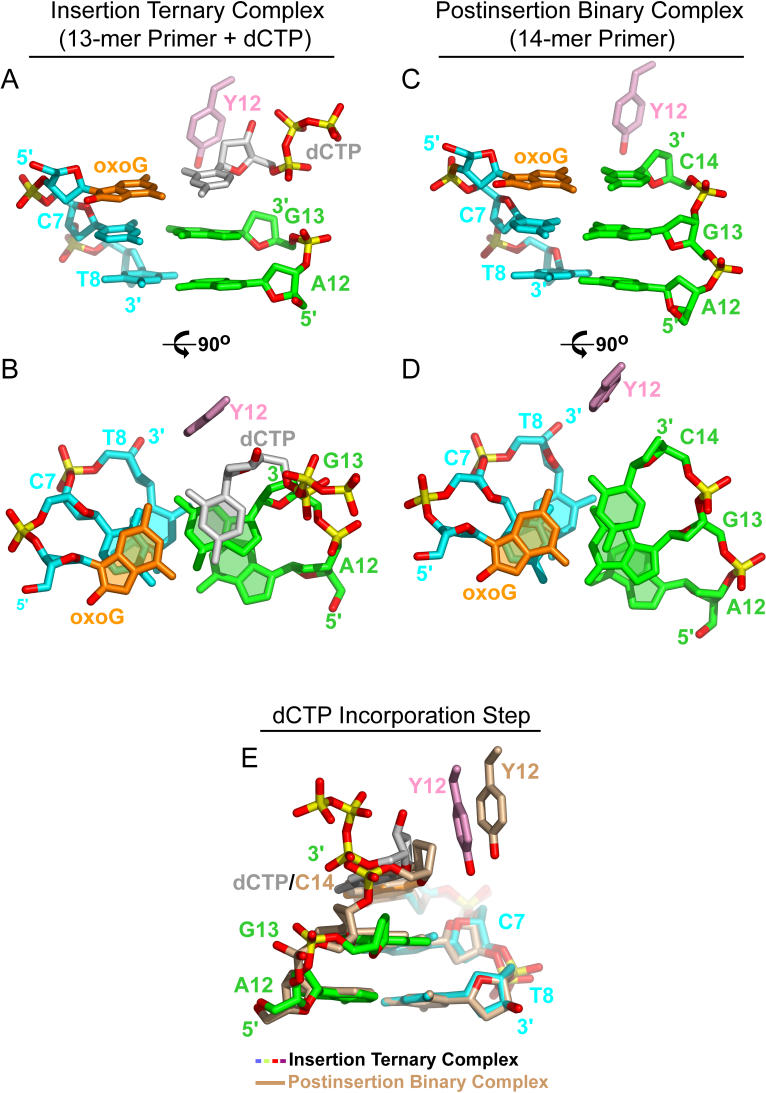
Relative Positioning of the oxoG•dCTP Pair in the Insertion Ternary Complex and the oxoG•C Pair in the Postinsertion Binary Complex (A) View of the (oxoG6-C7-T8)•(A12-G13) segment plus dCTP and Y12 in the modified ternary complex. The aromatic ring of Y12 is packed against the sugar ring of the incoming dCTP. (B) View of (A) looking down the helix axis. (C) View of the (oxoG6-C7-T8)•(A12-G13-C14) segment and Y12 in the postinsertion binary complex. (D) View of (C) looking down the helix axis. (E) Superposition of the (oxoG6-C7-T8)•(A12-G13) segment plus dCTP and Y12 of the modified ternary complex (in color) with the (oxoG6-C7-T8)•(A12-G13-C14) segment and Y12 of the posinsertion binary complex (in beige) by the oxoG6-C7-T8 and G6-C7-T8 template strands segments. Note the relative positioning of the sugar ring of dCTP/C14 and the aromatic ring of Y12 in the two complexes.

The position of the substrate, dCTP, with respect to the 3′-end nucleotide, G13, on the primer strand, differs substantially from a B-DNA step, with a reduction in the twist angle and displacement of the substrate base toward the DNA major groove (Figure 8A). The view looking down the helix axis clearly demonstrates this shifted alignment of dCTP relative to the 3′ portion of the primer strand (Figure 8B), with the sugar ring of dCTP positioned significantly closer to the sugar ring of G13, when compared with the typical B-DNA separation between A12 and G13. Upon covalent base incorporation, the restoration of the B-DNA conformation between the nascent base pair and its adjacent base pair (Figure 8C) propagates toward the 5′ end of the primer strand. When viewed down the helix axis, the separations between the sugar rings of G13 and the newly incorporated base, C14, and between G13 and A12, are approximately equal (Figure 8D).

The movement of primer strand 3′-terminal residue with respect to Dpo4 palm and finger domains is defined by the restraining function of the aromatic ring of Y12, which is packed against the sugar ring of the dCTP in the insertion ternary complex and against the sugar ring of newly incorporated C14 base in the postinsertion binary complex (Figure 8E). The conformational transition accompanying a restoration of the B-DNA step results in the contacts between the α-helical segments of the thumb domain and DNA phosphate backbone shifting by one step.

### Base-Pair Progression during dCTP Binding and Cytosine-Incorporation Steps

The DNA molecule retains a B-like conformation in the preinsertion binary, insertion ternary, and postinsertion binary complexes. However, there is a narrowing of the minor groove at the contact sites with the thumb domain and a widening of the major groove at the contact sites with the little finger, which is observed in all complexes, and which increases in the insertion ternary complex relative to both preinsertion binary and postinsertion binary complexes. The C7•G13 pair is adjacent to the oxoG lesion site, and its positioning within/adjacent to the Dpo4 active site is of interest in the three complexes.

**Figure 9:**
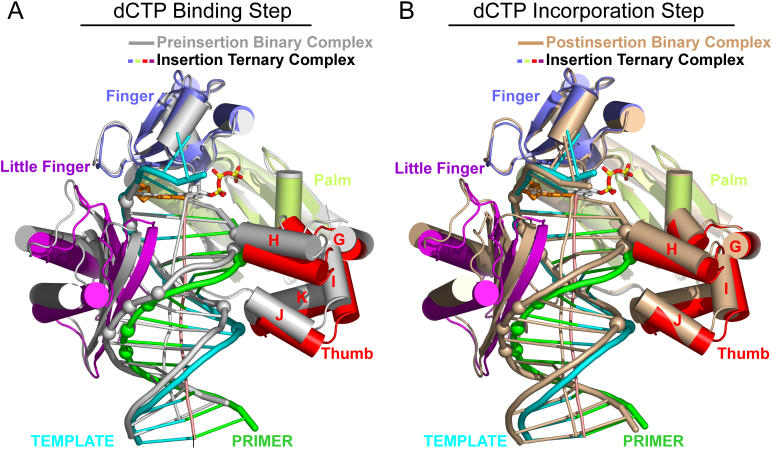
Conformational Transitions of Dpo4 and oxoG-Modified Template-Primer DNA Associated with Nucleotide Binding and Covalent Incorporation Steps Following Superimposition of Dpo4 Palm and Finger Domains (A) Overall comparative views (following superimposition of Dpo4 palm and finger domains) of the structures of the preinsertion binary complex (in silver) and the insertion ternary complex with incoming dCTP (in color). The DNA-backbone phosphate groups in contact with the Dpo4 little-finger and thumb domains are shown as spheres. Upon dCTP binding, DNA slides one step along the little-finger domain, and the oxoG residue of the template strand of the insertion ternary complex takes the place of C7 in the preinsertion binary complex. (B) Overall comparative views (following superimposition of Dpo4 palm and finger domains) of the structures of the insertion ternary complex with incoming dCTP (in color) with the postinsertion binary complex after covalent cytosine incorporation (in beige).

**Figure 10:**
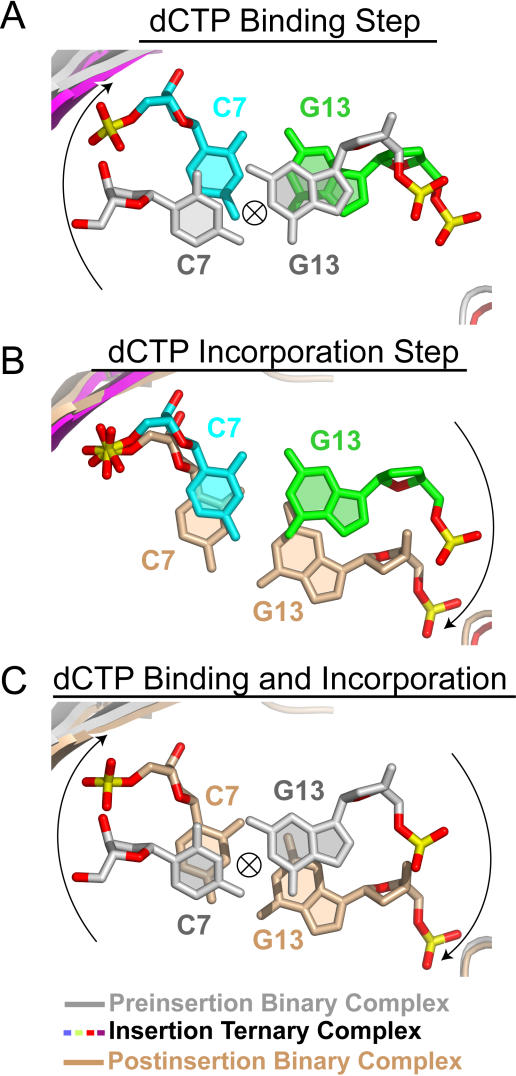
Comparison of the Relative Progression (Twist Angle Viewed down the Helix Axis) of C7•G13 Pairs between Complexes Superpositioned on their Finger and Palm Domains (A) C7•G13 pairs in the preinsertion binary (silver) and insertion ternary (color) complexes. (B) C7•G13 pairs in the insertion ternary (color) and postinsertion binary (beige) complexes. (C) C7•G13 pairs in the preinsertion binary (silver) and postinsertion binary (beige) complexes.

The preinsertion binary and insertion ternary complexes associated with dCTP binding can be compared by superpositioning their finger and palm domains, which constitute the Dpo4 active site (Figure 9A). The positioning of the C7•G13 pair relative to the active site for the two complexes is shown in Figure 10A. The template strand C7 of the preinsertion binary complex (in silver) is rotated and displaced along the helical axis to a new position in the insertion ternary complex (in color), while primer strand G13 is only displaced to maintain Watson-Crick hydrogen bonding, but is minimally rotated upon dCTP binding.

The insertion ternary and postinsertion binary complexes associated with dCTP incorporation can be compared by superpositioning their Dpo4 finger and palm domains (see Figure 9B). The relative positioning of the C7•G13 pair for the two complexes is shown in Figure 10B. Primer strand G13 of the insertion ternary complex (in color) is rotated and displaced to a new position in the postinsertion binary complex (in beige), while template strand C7 is only minimally displaced and rotated to maintain Watson-Crick hydrogen bonding.

Superposition of the preinsertion binary with the postinsertion binary complexes by their Dpo4 palm and finger domains (Figure S3A) results in almost ideal overlap between the DNA duplexes, with rotation and displacement with respect to the DNA helical axis by one full nucleotide step (Figure S3B). This can be best visualized by viewing the positioning of the C7•G13 pairs of the preinsertion binary (in silver) and postinsertion binary (in beige) complexes (Figure 10C).

## Discussion

**Figure 11:**
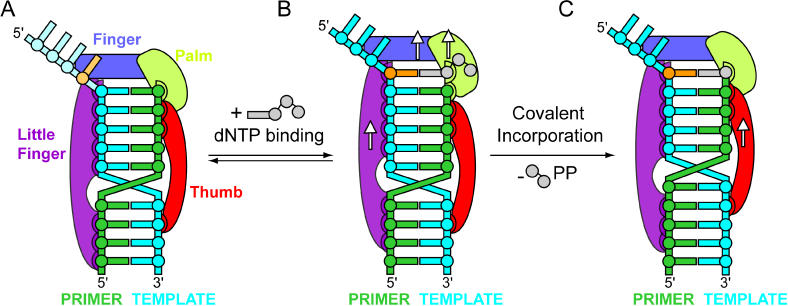
Schematic of Translocation Mechanism of Dpo4 Complexes with oxoG-Modified Template-Primer DNA (A) oxoG-modified preinsertion binary complex, where the overhang segment of the template strand, including oxoG, is disordered in the crystal (shown by light coloration). (B) oxoG-modified insertion ternary complex with incoming dNTP. (C) oxoG-modified postinsertion complex following covalent nucleotide incorporation. The arrows represent one nucleotide translocation of individual color-coded domains in the 3′-to-5′ direction of the template strand. Color-coding. DNA: template strand, cyan; primer strand, green; dCTP, light gray; oxoG residue, orange. Dpo4 domains: palm, light green; finger, blue; thumb, red; little-finger, purple; tether-connecting-thumb and little-finger, gray; Ca^2+^ cations, pink spheres.

The structures of the active sites of the preinsertion binary complex with no base opposite oxoG (see Figure 2D), the insertion ternary complex with incoming dCTP opposite oxoG (see Figure 2E), and the postinsertion binary complex following covalent C incorporation opposite oxoG (see Figure 2F) provide snapshots of the unique conformational changes of the Y-family Dpo4 polymerase as it translocates along the DNA molecule during one cycle of replication opposite the oxoG lesion (Figure 11).

### The dCTP-Binding Step: General Overview

The catalytic mechanism of Dpo4 involves movement of the bypass polymerase relative to DNA during both nucleotide binding (Figure 11A and 11B) and incorporation (Figure 11B and 11C) steps. Screw-like rotation of the polymerase counterclockwise with respect to the 5′-to-3′ direction of the template strand opens space for the next template residue to enter under the finger domain (movement of finger, palm, and little-finger domains shown schematically by arrows on blue, green, and purple backgrounds, respectively, in Figure 11B) and for the complementary incoming nucleotide to form a base pair with it. The incoming dNTP in the insertion ternary complex replaces the 3′ nucleotide in the preinsertion binary complex, since there is no open space to accommodate the entire incoming nucleotide, as in processive polymerases. The binding of dNTP could occur through initial positioning of its triphosphate, given that its binding site is the only part of the catalytic binding surface accessible prior to nascent base-pair formation. At this stage, the dimensions of the binding pocket discriminate against mispairs, while nascent base-pair formation involving complementary hydrogen-bond formation locks the template base and prevents it from sliding back out of the catalytic position.

The thumb and little-finger domains hold the DNA helix from opposite sides in bypass polymerase–DNA complexes and are the key determinants whose contacts most affect the relative rotation of the enzyme around DNA during dNTP-binding and nucleotide-incorporation steps. During the dNTP-binding step associated with nascent base-pair formation, the DNA is anchored to the thumb domain, and slides by one step along the little-finger domain (movement of little-finger domain is shown schematically by an arrow on purple background, Figure 11B). Since there are changes in the relative interdomain positions during this step, the helical axis of DNA is not a global axis for rotation of the complex. The bending in the DNA helical axis, due to additional narrowing of the DNA minor groove facing the thumb domain, and widening of the major groove facing the little-finger domain, help to accommodate the incoming nucleotide between the thumb and finger domains, while retaining thumb-domain–DNA contacts. The above outline describes the translocation and conformational changes associated with the first step and formation of a nascent base pair in a two-step sliding mechanism.

### Mechanisms of Nucleotide Selection by the Dpo4 Polymerase

Dpo4 imposes geometrical constraints on the length of the nascent base pair. From one side of the nascent base pair, the palm and finger domains define the position of the incoming nucleotide. There is shape-complementarity within the dNTP-binding pocket, because the dNTP sugar is packed against the aromatic side chain of tyrosine 12 and the triphosphate backbone with the chelated divalent cation precisely positioned within a channel lined by basic and acidic polar amino acids (see Figure 3B). From the other side of the nascent base pair, the little-finger domain holds the phosphate group of the template base. As a result, a longer purine–purine mispair would place the template base and incoming nucleotide phosphates farther apart, while a pyrimidine–pyrimidine mispair would bring them closer together. In both cases, the alignment of the active site in the ternary complex would be disrupted, including the placement of the second metal ion that accompanies the arrival of the dNTP at the binding site. The correct Watson-Crick base pair fits properly within the active site that is defined by the steric constraints that yield a ~10^−4^ error rate when an unmodified template strand is replicated. This selectivity is achieved despite the fact that the contour of the nascent base pair is such that its major and minor groove edges have minimal contacts with the surface of the bypass polymerase.

### The dCTP-Binding Step: Mechanistic Differences between Replicative and Bypass Polymerases

In replicative polymerases (Figure S4A), a dNTP binds to the open and accessible active site of a binary complex (Figure S4B) to form a base pair with the next available, unpaired template base *n*, with the O helix of the finger domain closing on the flat surface of the nascent base pair to form a tight-fitting, solvent-excluding, reaction-ready, ternary complex (Figure S4C) [16]. This conformational change is accompanied by a displacement of the template base *n* from a polymerase preinsertion pocket to a stacked position within the DNA helix [37]. A conserved tyrosine residue located at the base of the O helix facilitates this transition by vacating its stacking alignment with the (*n−1*) template base in the binary complex and occupying the polymerase preinsertion pocket in the ternary complex, thereby preventing a slippage of the next upstream template base into the active site. There are only minimal changes in the protein–DNA contacts beyond the base-pair recognition site during dNTP incorporation catalyzed by replicative polymerases.

By contrast, there is no open and accessible binding site for dNTP binding in the preinsertion binary complex of the Dpo4 bypass polymerase. Instead, the incoming dNTP must be inserted into the site previously occupied by the 3′-end primer residue. This position becomes accessible as a result of a rotating and sliding motion of the DNA along the little-finger domain by one nucleotide (see Figure 4C), while retaining phosphate contacts with the thumb domain (see Figure 4D), thereby placing the next template base (oxoG or unmodified G) in a stacked conformation under the finger domain. The binding of dCTP locks this position, orienting it for the subsequent nucleotidyl transfer reaction. There are no local conformational changes within the finger domain when dCTP binding occurs, and, therefore, there is no transition from an open to a closed complex in this bypass polymerase, since the finger domain does not undergo any conformational changes.

### The Cytosine-Incorporation Step: General Overview

The incoming dNTP, whose base is displaced toward the major groove and exhibits a substantially reduced twist angle, is inclined such that its catalytically competent phosphate oxygen is poised for coordination with both the 3′-end nucleotide of the primer strand and divalent cations (see Figure 8A and 8B). The conformational change associated with the transition from the underwound position of the bound dNTP to the restored B-DNA geometry on phosphodiester bond formation (see Figure 8C and 8D) is propagated bidirectionally, perhaps analogous to the release of a compressed spring. There is an increased separation between the sugar ring of dNTP, which is packed against and restrained by the aromatic ring of Tyr12, and the sugar ring of the 3′-end nucleotide of the primer strand, on phosphodiester bond formation (see Figure 8E), thereby initiating the translocation of the α-helical contacts of the thumb domain by one step along the phosphodiester backbones of both primer and template strands (movement of thumb domain is shown schematically by an arrow on red background, Figure 11C). The above outline describes the translocation and conformational changes associated with the second step, covalent bond formation, in a two-step sliding mechanism.

### The Cytosine-Incorporation Step: Mechanistic Differences between Replicative and Bypass Polymerases

Phosphodiester bond formation in replicative polymerases releases pyrophosphate, thereby breaking apart the triphosphate backbone-mediated interactions between the palm and finger domains. This results in the movement of the O helix of the finger domain back to the open state, while positioning the aromatic ring of a tyrosine for stacking over and pushing against the newly formed base pair, thus triggering translocation. The polymerase translocates upward by one base-pair step in the 3′ to 5′ direction of the template strand, and the minor groove edge of the nascent base pair is examined for correct Watson-Crick pairing alignment by the proofreading apparatus of the polymerase [16,37].

In the case of Dpo4, release of the pyrophosphate is not accompanied by breaking apart the triphosphate backbone-mediated interactions between the palm and finger domains. The formation of the phosphodiester bond instead triggers rotation of the entire DNA molecule relative to the polymerase active site, formed by the finger and palm domains, in order to create a greater distance and thus diminish repulsive interactions involving phosphate groups between the newly incorporated C residue and the adjacent nucleotide on the primer strand. As a result, the contacts between the thumb domain and the backbone DNA phosphate groups shift by one nucleotide step (see Figure 7D). The nucleotidyl transfer reaction and release of the pyrophosphate provide the driving force for this transition. The nascent base pair retains its contacts with the finger domain (see Figure 7C), and there are no proofreading interactions between this base pair and the Dpo4 polymerase.

The structures of the unmodified preinsertion binary and insertion ternary Dpo4 complexes are identical to their oxoG-modified counterparts with the exception of a different location of the oxoG phosphate group and Arg 332 in the insertion ternary complex (see Figure 3F). Thus, the above-described mechanism of dNTP binding to the preinsertion binary complex to form the insertion ternary complex applies not only in the case of the oxoG-lesion but also to unmodified DNA templates.

### Structure-Based Mechanistic Insights into Dpo4 Insertion Accuracy Opposite the oxoG Lesion and Comparison with Replicative Polymerases

Dpo4 and the Y-family yeast and human pol η [32] are able to achieve a 100-fold preference for inserting dCTP over dATP opposite oxoG. However, replicative T7 [38] and Rb69 [18] incorporate dCTP only two to seven times more frequently than dATP [18,34,38], the gap-filling pol β has a slight preference for inserting dATP [39], while replicative pol α, pol δ, *Bacillus* pol I, and HIV-1 reverse transciptase predominantly insert dATP [29,35,38,39].

The polymerases pol β, Rb69, T7, and Dpo4 avoid the steric clash of the O8 atom with the sugar-phosphate backbone that arises in the oxoG*(anti)* •dCTP base pair either by a sharp kink in the single-stranded template overhang within Rb69 [18] and T7 polymerase [34] active sites, or, in the case of Dpo4 (this study) and pol β [33], by a flipping of the phosphate group of oxoG by 180°. However, Dpo4 forms six hydrogen bonds with oxoG*(anti)* in the active site of Dpo4 (five with the phosphate group of oxoG and one with the O8 atom through a water bridge, see Figure 3D), while pol β [33] and Rb69 [18] do not make any specific contacts with oxoG*(anti),* and T7 forms only one hydrogen bond with O8 of oxoG by the relocated side chain of a lysine residue [34]. Thus, the multiple and favorable contacts of oxoG*(anti)* with amino acids within the Dpo4 active site appear to be the most important factor responsible for the remarkable fidelity of replication of oxoG lesion catalyzed by Dpo4.

The uniqueness of the Dpo4 base-insertion mechanism among the polymerases studied in detail provides a second factor for the preferred incorporation of dCTP opposite oxoG. We hypothesize that, prior to dNTP binding, the next available template base to be replicated interconverts between a looped-out conformation in the single-stranded overhang and a base–base stacking conformation involving the 3′ base in the template strand. Once the oxoG base becomes inserted into the helix in this manner, we hypothesize that the more favorable contacts of amino acid residues in Dpo4 with oxoG*(anti)* rather than with oxoG(*syn*) drive the *(anti)–(syn)* equilibrium toward the *anti* conformation.

By contrast, the mechanism of replicative polymerases, as well as gap-filling pol β, do not allow any control over the conformation of oxoG before nucleotide binding. In Rb69, T7, and *Bacillus* pol I, the next to-be-replicated template base *n* is prevented from stacking into the helix by the conserved tyrosine at the base of the O helix [37]. This tyrosine is displaced out of its position by the template base *n* in the same timeframe as formation of a base pair with an incoming dNTP. An incoming dCTP favors base-pairing with oxoG in the *anti* conformation (Figure S5A), while an incoming dATP would facilitate pairing with oxoG in the *syn* conformation (Figure S5B)*,* both occurring before the O helix is closed and the active site is fully assembled. Thus, the conformation of the oxoG residue would be selected by its base-pairing partner. In the pol β binary complex with a single-nucleotide gapped substrate [40], the next template base *n* is stacked into the helix. Thus, the oxoG conformation might be already fixed even before the dNTP is bound. However, neither the phosphate group of the oxoG residue [33], nor the unmodified G [40], form any contacts with pol β. Apparently, in the case of pol β, as well as T7, Rb69, and *Bacillus* pol I, there is no mechanism available that can influence potential *(anti)–(syn)* equilibrium of oxoG before dNTP is bound, as is the case in Dpo4.

Dpo4 demonstrates the ability to achieve at least 10-fold higher accuracy of insertion of dCTP opposite oxoG than the high-fidelity T7 and Rb69 polymerases. This is achieved by imposing an *anti* conformation on the oxoG lesion that, in turn, favors pairing with dCTP, thus resulting in a non-mutagenic bypass of oxoG catalyzed by Dpo4. Furthermore, the binding of dCTP further stabilizes oxoG*(anti)* due to a higher thermodynamic stability of the oxoG*(anti)* •dCTP (Figure S5A) over the oxoG*(syn)* •dATP (Figure S5B) mispair at the template-primer junction [41].

### Conclusions

The outlined research allows us to draw three major conclusions that add to our current understanding of lesion bypass by Y-family Dpo4 polymerase.

First, two arginine residues (R331 and R332) of Dpo4 play a critical role in accommodating the oxoG lesion and facilitating an *anti* conformation for Watson-Crick base pairing with dC.

Second, in the stepwise nucleotide incorporation opposite an oxoG, reflecting transitions from preinsertion binary to insertion ternary, and subsequently to postinsertion binary complexes, the little-finger and thumb domains move one at a time, tracking the template and primer strand translocation separately.

Third, the dCTP bound in the insertion stage is not properly base-stacked with the DNA duplex as in regular B-form DNA. This suggests that the ternary complex may need to undergo subtle conformational changes prior to the chemistry step of phosphoryl transfer.

## Materials and Methods

### Preparation and purification of template and primer DNA strands

The DNA 19-mer template 5′- CTAAC[G*] CTACCATCCAACC-3′ with [G*] = oxoG or G, with a 3′-OH terminus was synthesized using an automated Applied Biosystems (Foster City, California, United States) 392 DNA synthesizer with phosphoramidite chemistry, cleaved from the support, deprotected with ammonium hydroxide for 5 h at 55 °C (with 0.25 M 2-mercaptoethanol for 17 h for the oxoG-modified oligonucliotide to avoid any oxidative degradation of the oxoG site), purified on 20% polyacrylamide gel in the presence of 8 M urea, electro-eluted, and desalted. 2′,3′-dideoxy-G at the 3′ end was introduced into the 13-mer primer 5′- GGTTGGATGGTAG-3′ by reverse 5′-to-3′ synthesis using 5′-CE phosphoramidites, the 14-mer primer with 2′,3′-dideoxy-C-3′ terminal was synthesized using 2′,3′-ddC columns by regular 3′-to-5′ synthesis, and purified as described. All phosphoramidites were purchased from Glen Research (Sterling, Virginia, United States). [γ−^32^P]ATP (specific activity, >3000 Ci/mmol) was obtained from NEN LifeScience Products (Perkin-Elmer, Boston, Massachusetts, United States), T4 polynucleotide kinase was purchased form Amersham Pharmacia Biotech (Piscataway, New Jersey, United States), and the reagents for crystallization were obtained from Hampton Research (Aliso Viejo, California, United States).

### Preparation and purification of Dpo4

The DNA fragments encoding 353 amino acid full-length Dpo4 (plus 5 glycines at the N-terminus) were obtained by PCR and inserted into pET-28a between NdeI and EcoRI cleavage sites. The His-tag version of Dpo4 was expressed in *E. coli* BL21–CodonPlus (DE3)-RIL strain (Stratagene, La Jolla, California, United States), and then subjected to a Ni-chelating column. The 6-His tag was next cleaved with thrombin, and the Dpo4 protein was then further purified by heparin column chromatography, followed by passage through a Superdex-200 column. Dpo4 was concentrated to 26 mg/ml in 5 mM DTT, 25 mM HEPES (pH 7.5), and 300 mM NaCl.

### Primer elongation standing-start assay

The template-primer DNA complexes formed by annealing a 19-mer template containing oxoG (or G at the same site) with a ^32^P 5′-end-labeled 3′-OH terminated primer (13-mer ending one base before the oxoG site, or 14-mers with C or A opposite G or oxoG), were incubated with Dpo4 polymerase in the presence of all four dNTPs. Aliquots were withdrawn from the reaction mixture after 1, 3, 5, 10, 15, and 20 min incubation times and quenched by a gel-loading buffer (95% formamide with 20 mM EDTA, 45 mM Tris-borate, 0.1% bromophenol blue, 0.1% xylene cyanol). For a typical experiment, a 12-μl solution of all four dNTPs was added to the 12-μl polymerase plus template-primer DNA mixture, both solutions were in 100 mM HEPES (pH 8.0), 5 mM MgCl_2_, 60 mM NaCl, and 5 mM dithiothreitol. The polymerization reactions were conducted at 30 °C, final template-primer DNA concentration was 10 nM, Dpo4 was 1 nM, and each dNTP 100 μM. The reaction products were resolved on 20% polyacrylamide gels in the presence of 8 M of urea, and the gels were dried before radiography with Fuji image plate (Fuji Photo Film Company, Tokyo, Japan). The images were scanned on Fuji PhosphoImager (Fuji), and the bands were quantified using profile analysis mode in ImageGauge software (Fuji).

### Steady-state kinetic analysis of one-base insertion and extension

Steady-state kinetics parameters were analyzed for incorporation of each deoxynucleotide opposite the G and oxoG and for extension of primers with C or A 3′-terminal opposite G and oxoG in the presence of the next correct nucleotide, 2′-deoxyguanosine 5′-triphosphate (dGTP), as described in [42]. In a typical experiment, 6-μl solutions with increasing concentrations of single dATP, dTTP, dGTP, or dCTP were added to 6 μl of polymerase/template-primer DNA mixture, and the reactions were stopped after 3 min by the addition of 12 μl of denaturing loading buffer. To ensure single-hit polymerization conditions (less than 20% primer extended), the nucleotide concentration interval was adjusted for every experiment. The template-primer DNA concentrations were 50 nM, and Dpo4 was 1 nM. The gel-band intensities of extended and unreacted primer strands were quantified as described. The reaction rates (ν, nM/min) were plotted as a function of the dNTP concentration, and the data were fit by nonlinear regression of the Michaelis-Menten equation,



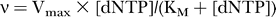



to calculate apparent K_M_ and V_max_ steady-state parameters [32,42]. The frequency of nucleotide incorporation (f_inc_) and extension (f_ext_) were defined as follows:



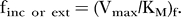



### Crystallization

The crystals of the Dpo4 ternary complexes containing oxoG (or G) 19-mer templates and the 13-mer primer terminated by 2′,3′-dideoxyguanosine were grown in the presence of dCTP. The preinsertion binary complexes in the absence of dCTP, the postinsertion binary complex with the oxoG-modified template, and the 14-mer primer 3′-terminated with 2′,3′-dideoxycytosine were grown under conditions similar to those described in the literature [5]. Briefly, template-primer DNAs were annealed and mixed with Dpo4 in 1.2:1 molar ratio to a final concentration 0.15 mM in 20 mM HEPES (pH 7.0), 60 mM NaCl, 5 mM MgCl_2_, 1mM DTT, and dCTP (1 mM) was added to produce the ternary complexes. The protein-DNA complexes were then incubated at 37 °C for 5 min and centrifuged at 11,000 rpm for 7 min at 4 °C. The crystals were grown by the hanging-drop method against a reservoir solution containing 100 mM HEPES (pH 7.0), 100 mM calcium acetate, and 10% PEG 4000 at 20 °C. Several rounds of micro seeding (Hampton Research kit) were employed to produce the diffraction quality crystals of the short binary complexes. The crystals were transferred to a cryosolution containing the mother liquor with 15% PEG 4000 and 15% ethylene glycol and flash-frozen in liquid nitrogen for X-ray data collection.

When compared with the type I structure determined by Yang and colleagues [5], the little-finger domain in both of our oxoG-damaged and unmodified ternary complexes is shifted by up to 0.5 Å and rotated by 5° toward the finger domain, leading to a concomitant shift of the DNA duplex. However, the contacts with the equivalent phosphate groups of the DNA backbone are maintained. This repositioning of the little finger is likely due to the more spacious crystal lattice with a 10% higher solvent content and the different DNA sequence in our case. Similar rearrangements of the little-finger domain were noticed for crystals of ternary complexes characterized by different lattices [12]. Thus, *RMSD* of polymerase Cα atoms between our oxoG**-**modified, and the unmodified ternary type I complex reported previously [5], is only 0.368 Å, excluding the little finger, and 0.667 Å for all 341 residues.

### Structure determination and refinement

X-ray diffraction data were collected either at an Advanced Photon Source 14-ID beam line (Argonne National Laboratory, Chicago, United States) or with an in-house Rigaku R-axis IV detector (Rigaku, The Woodlands, Texas, United States) mounted on an RU 200 generator. The data were processed and scaled using HKL2000 suite (Structural Biology Center, Argonne National Laboratory). The crystal of the oxoG-modified insertion ternary complex with incoming dCTP belonged to the P2_1_ space group, and exhibited unit cell parameters not observed previously for Dpo4-DNA complexes [5,10–13]. The structure was solved by molecular replacement (AMoRe [43]) using Dpo4-DNA-ddADP type I structure [5] as a search mode. The little-finger domain (residues 244–341) was split from the complex and its position refitted by AMoRe. The model building, including substitution of the DNA sequence, was manually finished in TURBO based on the electron density map calculated in X-PLOR, and the resulting model was refined in REFMAC [44] at 1.95 Å resolution to a final *R_factor_*/*R_free_* of 0.226/0.253 (see Table 2).

The structure of the oxoG-modified preinsertion binary complex was solved by molecular replacement in the P2_1_ space group (see Table 2), employing refined oxoG-modified insertion ternary complex as a search model. After the initial positions of both molecules in the asymmetric unit cell were defined, the model was split into Dpo4 domains (the palm-finger, little-finger, and thumb) and the DNA duplex, and the new positions of the thumb and little-finger domains were found. The template-primer DNA was retraced in TURBO based on the electron density map produced by CNS after rounds of refinement with the rigid group, rigid pair, and annealing routines in CNS. The crystal displayed pseudo-merohedral twinning [45] with twin fraction of 0.4, as estimated from the Britton plot generated by the DETWIN module of CCP4. The data were detwinned (Protocol S1), and the model was refined in REFMAC at 2.35 Å resolution to *R_factor_*/*R_free_* of 0.249/0.318 (see Table 2). Despite the relatively high *R*-factors (probably a consequence of twinning), the final electron-density map clearly showed molecular details.

Molecular replacement with the oxoG-modified insertion ternary complex as a search model was used to solve the structure of the oxoG-modified postinsertion binary complex with C opposite the lesion site. The final structure was refined by REFMAC to 2.65 Å and *R_factor_*/*R_free_* of 0.238/0.287 (see Table 2).

The structures of the unmodified insertion ternary complex with incoming dCTP and preinsertion binary complexes with primers terminated with dideoxy- and deoxyguanine were solved by molecular replacement employing the structures of oxoG-modified ternary and binary complexes as search models, and refined by REFMAC to values of *R*-factors indicated in Table 2. Detwinning in the case of an unmodified preinsertion complex did not improve refinement statistics. However, careful processing of the data set allowed us to refine the structure to *R_factor_*/*R_free_* 0.271/0.310 (see Table 2).

### Structure analysis

Dpo4 conformations in the ternary and binary complexes were compared by the program DynDom to determine protein domains, hinge axes, and amino acid residues involved in the hinge bending [46]. Parameters of DNA duplexes were calculated by the program Curves*.*

## Supporting Information

Figure S1 Structures of Complexes of Dpo4 with oxoG-Modified Template-Primer DNA (A) Overall structure of the preinsertion binary complex. Template residues 1–6 (including oxoG) of single-stranded overhang are disordered in the electron density maps.
(B) Overall structure of the insertion ternary complex, with incoming dCTP paired with oxoG*(anti)* at the active site.
(C) Overall structure of the postinsertion binary complex, with covalently incorporated C paired with oxoG*(anti).*
The DNA and protein are in stick and ribbon representations, respectively. Note that there is a 2′,3′-dideoxy residue at the 3′ end of the primer strand in all three complexes. Color-coding for top lane. DNA: template strand, cyan; primer strand, green; dCTP, light gray; oxoG residue, orange. Dpo4 domains: palm, light green; finger, blue; thumb, red; little-finger, purple; tether-connecting-thumb and little-finger, gray; Ca^2+^ cations, pink spheres.
(6.3 MB TIF).

Figure S2 Structures of Complexes of Dpo4 with Unmodified Template-Primer DNA (A) Overall structure of the preinsertion binary complex. The DNA and protein are in stick and ribbon representations, respectively. Template residues 1–6 (including G) of single-stranded overhang are disordered in the electron density maps.
(B) Overall structure of the insertion ternary complex with incoming dCTP.
The DNA and protein are in stick and ribbon representations, respectively. Note that there is a 2′,3′-dideoxy residue at the 3′ end of the primer strand in both complexes. Color-coding for top lane. DNA: template strand, cyan; primer strand, green; dCTP, light gray; oxoG residue, orange. Dpo4 domains: palm, light green; finger, blue; thumb, red; little-finger, purple; tether-connecting-thumb and little-finger, gray; Ca^2+^ cations, pink spheres.
(C) Details of the active site of the preinsertion binary complex.
(D) Details of the active site of the insertion ternary complex with incoming dCTP.
Note that there is a 2′,3′-dideoxy residue at the 3′ end of the primer strand in both complexes.
(6.6 MB TIF).

Figure S3 Structural Equivalence of Preinsertion and Postinsertion oxoG-Modified Binary Complexes (A) Overall comparative views (following superimposition of Dpo4 palm and finger domains) of the structures of the preinsertion binary complex (in silver) and the postinsertion binary complex (in beige). The DNA-backbone phosphate groups in contact with the Dpo4 little-finger and thumb domains are shown by spheres, in silver for preinsertion and in beige for postinsertion complexes.
(B) Top five DNA duplex base pairs (following superimposition of Dpo4 palm and finger domains) of the preinsertion binary complex (in silver) and the postinsertion binary complex (in beige). Upon completion of a Dpo4 catalytic cycle, the DNA duplex is shifted one full base-pair step, so that the template strand residues from oxoG to C10 of the postinsertion complex take the place of C7 to C11 in the preinsertion binary complex, while primer strand C14 to G10 of the postinsertion complex takes the place of G13 to G9 of the preinsertion complex. The DNA-backbone phosphate groups in contact with the Dpo4 little-finger and thumb domains are shown by transparent spheres.
(4.4 MB TIF).

Figure S4 Conformational Changes in pol I Family Klentaq Polymerase upon Nucleotide Binding Involving Transition from an Open Binary to Closed Ternary Complex (A) Overall comparative view (following superimposition of palm domains) of the structures of the open binary complex (in silver) (PDB ID: 4KTQ), and closed ternary complex with a bound dCTP (in color) (PDB ID: 3KTQ) [S2]. On dNTP binding, the finger domain (blue in ternary complex) rotates as a rigid body by 6° toward the palm domain (light green in the ternary complex), while the O helix (colored light yellow in the binary complex and bright yellow in the ternary complex) moves by 40° to form the closed ternary complex.
(B) The Klentaq active site in open binary complex.
(C) The Klentaq active site in closed ternary complex.
(6.4 MB TIF).

Figure S5 Base-Pairing Alignments of 8-oxo-Guanine (oxoG) (A) Hydrogen-bonding alignments in oxoG*(anti)*•C*(anti)* pair.
(B) Hydrogen-bonding alignments in oxoG(*syn*)•A*(anti)* pair.
(477 KB TIF).

Protocol S1 Experimental Procedures: Full Description of the Twinning Problem for Preinsertion Binary Complexes (28 KB DOC).

### Accession Numbers

Coordinates for the oxoG-modified binary preinsertion (2ASJ), ternary insertion (2ASD), and binary postinsertion (2ASL) complexes; and unmodified binary preinsertion (2AU0); and ternary insertion (2ATL) complexes have been deposited in the Protein Data Bank (http://www.rcsb.org/pdb).

## Abbreviations

dATP: 2′-deoxyadenosine 5′-triphosphate
dCTP: 2′-deoxycytidine 5′-triphosphate
dNTP: deoxyribonucleotide triphosphate
Dpo4: DNA polymerase IV
G: guanine
oxoG: 8-oxoguanine
RMSD: root mean square deviation
