# Supplementary material for: Stepwise Translocation of Dpo4 Polymerase during Error-Free Bypass of an oxoG Lesion
Source: PLoS Biol. 2006 Jan 3;4(1):e11. doi: 10.1371/journal.pbio.0040011 (PMC1325099; doi:10.1371/journal.pbio.0040011)
Supplement: Supplementary file 1 [file pbio.0040011.sd001.doc]

**Experimental Procedures**

**Full Description of the Twinning problem for Preinsertion Binary Complexes**

Both for oxoG-modified and unmodified presinsertion binary complexes crystals belong to monoclinic P2(1) space group with two unit cell parameters *a* and *c* equal to each other, which allows for a rare type of pseudo-merohedral twinning [S1]. The twinning operator in pseudo-merohedral twins introduces additional symmetry that suggest a higher Laue symmetry than is possible for a monoclinic space group, so the two unit cells of the twin domains share the same *a* and *c* axes, but with opposite directions. The presence of twinning is known to complicate refinement. Since, we successfully solved the structure of the oxoG-modified preinsertion binary complex by molecular replacement in P2(1) space group, which normally does not allow twinning, we disregarded an earlier observed indicator of possible twinning that our data could be indexed, processed and scaled with comparable R_merge_ values in lower symmetry monoclinic P2(1) and higher symmetry orthorhombic C222(1) space groups. Thus, the analysis of our data with DETWIN module of CCP4 estimated twin fraction for oxoG-modified preinsertion complex to be around 0.4 (from the Britton plot), and 0.32 for unmodified complex. The twin operator l, -k, h describes a real space rotation necessary to superimpose the cell with vectors *a, b, c* and oppositely directed cell with vectors c, -b, a. Refinement of oxoG-modified presinsertion complex against detwinned data let us improve R_factor_/R_free_ to 0.249/0.3175 (instead of 0.260/0.330) with the same test data for cross-validation, although we have not been able to resolve twining problem entirely. Detwinning in the case of unmodified preinsertion complex did not improve refinement statistics. However, reprocessing of the data set for the unmodified preinsertion complex allowed us to refine the structure to R_factor_/R_free_ 0.271/0.310 (instead of 0.289/0.389). We should mention that the electron density maps calculated previously with twinned data for oxoG-modified and unmodified structure were clear with well defined molecular details despite on high R_factor_ and R_free_ . Detwinning and data reprocessing did not visibly improve the maps. It is likely that the value of R_free_ have not been estimated correctly, as the test set is not entirely independent of the working set due to the presence of pseudo-symmetry operator.

**Supplemetary References**

S1. Rudolph MG, Wingren C, Crowley MP, Chien YH, Wilson IA (2004) Combined pseudo-merohedral twinning, non-crystallographic symmetry and pseudo-translation in a monoclinic crystal form of the gammadelta T-cell ligand T10. Acta Crystallogr D Biol Crystallogr 60: 656-664.

S2. Li Y, Korolev S, Waksman G (1998) Crystal structures of open and closed forms of binary and ternary complexes of the large fragment of Thermus aquaticus DNA polymerase I: structural basis for nucleotide incorporation. Embo J 17: 7514-7525.
